# Forecasting Slope Displacement of the Agricultural Mountainous Area Based on the ACO-SVM Model

**DOI:** 10.1155/2022/2519035

**Published:** 2022-09-29

**Authors:** Juan Chen, Yiliang Wei, Xiaohui Ma

**Affiliations:** ^1^Shanxi Vocational University of Engineering Science and Technology, Taiyuan 030031, Shanxi, China; ^2^Taiyuan Design Institute of Railway Engineering Consulting Group Co Ltd, Taiyuan 030000, Shanxi, China

## Abstract

Due to the combined influence of complex engineering geological conditions and environmental factors from agricultural mountainous areas, the evolution of slope deformation is complicated and nonlinear. Support vector machine (SVM) technology could effectively solve the technical problems of small sample, high dimension, and nonlinear, so it is applied to data mining of the measured slope displacement and the prediction and analysis of the slope deformation trend. In order to avoid blindness of human choice of SVM parameters and to improve the prediction accuracy and generalization ability of the model, an ACO-SVM model is built by adopting an improved ant colony algorithm (ACO) to optimize parameters in association with the rolling forecasting method of displacement time series. The model was applied to two engineering examples. The research results showed that the ACO-SVM model was correct with high accuracy. The ACO-SVM model had higher accuracy of prediction and stronger generalization ability than optimizing SVM based on the genetic algorithm or particle swarm optimization. The forecasting results were more reasonable. It has certain engineering application values for slope deformation prediction.

## 1. Introduction

Landslides are one of the most harmful natural disasters in the world and pose a serious threat to human beings and society. Monitoring and early warning of the slope displacement is the main technical means to avoid this hazard [1]. According to the displacement monitoring data, the future evolution and regular development trend of the slope will be predicted. It is of great significance to grasp the slope deformation law for engineering management in the future. At present, the prediction methods of displacement of time series mainly include time series analysis, grey system, artificial neural network, support vector machine (SVM), and various combinations of prediction methods [2]. In all methods, time series analysis is difficult to determine the parameter of an autocorrelation coefficient *r*, a partial correlation coefficient *q*, and reasonable identification to the model. The grey system is mainly suitable for the time series of exponentials, and the prediction results often have large errors for complex nonlinear slope displacement series [3]. An artificial neural network is based on the heuristic algorithm, and its disadvantage is that there is no complete theoretical basis. When the number of samples is limited, it is difficult to guarantee the accuracy, while when there are many learning samples, it is easy to fall into the dimension disaster, and the generalization ability of the algorithm is not high. A support vector machine (SVM) based on the principle of structural risk minimization has strong generalization ability and effectively solves problems such as small sample, high dimension, and nonlinear. SVM can realize adaptive decomposition according to the data scale and obtain the displacement of the trend term, periodic term, and random term by setting a static component, which has the advantages of high decomposition precision, strong robustness, and clear physical meaning.

One of the outstanding problems of SVM is that it is difficult to determine the parameters of penalty parameter C and kernel function *γ* and the accuracy of SVM is directly related to the ability of model prediction and generalization. Previous studies have conducted a lot of research on the optimization of SVM model parameters, mainly involving metaheuristic algorithms, such as the simulation of the living habits of the American monarch butterfly, and the monarch butterfly optimization (MBO) [4] migration operator biased to local search. A slime mould algorithm (SMA) [5] was proposed based on the oscillating predation behavior of slime mould individuals. Proposed by Al-Attar Ali Mohamed in 2016, a moth swarm algorithm (MSA) [6] was designed inspired by the photo taxis and flight patterns of moths. Hunger games search (HGS) [6] based on animal hunger-driven activities and behavior was proposed. The Runge–Kutta method (RUN) is a high-precision single-step algorithm widely used in engineering. Kok Meng of Malaysia proposed the swarm predation algorithm (CPA) [6] in 2020, inspired by how carnivorous plants adapt to harsh environments such as insect hunting and pollination. The weighted mean of vectors algorithm (INFO) is a new intelligent optimization algorithm proposed in 2022, which achieves optimization through different weighted average rules of vectors. Harris hawks optimization (HHO) [7] is a swarm optimization algorithm proposed by Heidari in 2019, which simulates the predation behavior of Harris hawks. Ant colony optimization (ACO) is a new method to simulate biological evolution and has the advantages of parallel computing, positive feedback searching, and good adaptability [8]. The feature parameter dimension of parameter data in an SVM model is higher. In order to avoid “dimension disaster,” overfitting phenomenon, and improve the classification accuracy and efficiency of the model, feature selection is necessary. Feature selection is essentially a combinatorial optimization problem, so an ACO algorithm can be used to reduce dimension.

In this paper, the improved ant colony optimization algorithm is proposed on the basis of existing research, and support vector machine (SVM) technology is used to optimize the prediction model so as to reduce the blindness of model parameter selection. This technique is applied to slope monitoring, displacement training and learning, and rolling prediction of displacement change time sequences [9]. In this paper, several commonly used algorithms and the improved ACO-SVM are applied to slope treatment technology, and the efficiency of the algorithm is analysed and compared; in the end, the superiority of the improved ACO-SVM is verified. The specific research work includes the following three aspects: (1) monitoring the changes of horizontal displacement data of slope of a hydropower station and determining monitoring parameters; (2) based on the improved ACO-SVM algorithm model, the slope stability is analysed; (3) the slope displacement is predicted and analysed based on the improved algorithm model. The improved ACO-SVM model has higher prediction accuracy, stronger generalization ability, and more reasonable prediction results, which has certain engineering application values in slope deformation prediction.

## 2. Materials and Methods

### 2.1. Research Status of Slope Deformation

In recent years, slope deformation accidents caused by engineering construction, earthquakes, and rainfall have occurred in many places in China. Many scholars in the world have performed relevant work. For example, a GM (1, 1) model was successfully applied to the north slope of a tunnel by using the grey system model for beneficial exploration [10]. Combining the relevant scientific knowledge with traditional grey theory, the unbiased grey fuzzy Markov prediction model is constructed and applied to the slope displacement prediction of a mine. The least-squares support vector machine and radial basis kernel function were used to train and predict slope data, and the optimal model parameters were found by a quantum particle swarm optimization algorithm [11].The results show that the prediction accuracy is improved. Numerical simulation and slope stability analysis were used to evaluate and predict the high slope stability of tailings. The Gaussian kernel function and polynomial kernel function were combined to construct the mixed kernel function, and the particle swarm optimization algorithm was applied to find the best parameters of the least-squares support vector machine [12]. Finally, the PSO-LSSVM model of the slope displacement sequence was established, and it was applied to the left bank slope data of a hydropower station [13]. After the slope stability parameters were determined, the limit equilibrium method and discrete element numerical simulation were adopted to analyse the stability and failure mode of the open-pit mine slope [14, 15]. Considering six influencing factors of the research object, the slope stability of an open-pit mine could be judged and calculated by gene expression. Tan et al. proposed a new method to measure slope stability, which is to calculate the permanent displacement of slope by the stiffness reduction method [16].

It can be seen that there are many factors affecting slope deformation, and some models have to take many factors into account in slope prediction, which undoubtedly increases the difficulty of slope deformation prediction [17].

### 2.2. Support Vector Machine Regression Prediction Model

SVM is a small sample intelligent learning algorithm proposed by Vapnik based on statistical theory. This algorithm uses kernel snapping from transforming the known space to higher dimensional space through nonlinear mapping so that the samples of higher dimensional space could be linearly separable [3].

For the regression problem, let the training sample set be expressed as {(*x*_*i*_, *y*_*i*_)|*x*_*i*_ ∈ *R*_*d*_, *I* = 1, 2, ..., *n*}, *x*_*i*_ is the d-dimensional vector of input, *y*_*i*_ is the output value, *R* is the set space of all real numbers, and *n* is the number of samples. Make nonlinear mapping: *R*_*d*_ ⟶ *H*, where is a high-dimensional feature mapping and *H* is a high-dimensional feature space, and we construct the optimal decision function in this feature space, the formula is as follows:

(1) $$\begin{array}{c} y\left(x\right)=w\varphi \left(x_{i}\right)+b\text{,} \end{array}$$

In the formula, *w* is the weight vector in an *H* space and *b* is the offset term. The fitting error and the insensitive function ε are considered, and the relaxation factors and are introduced to minimize the objective function (2) of the error.

(2) $$\begin{array}{c} J\left(x_{i},\xi_{i},\xi_{i}^{*}\right)=\frac{1}{2}w.w^{T}+C\overset{n}{\underset{i=1}{\sum }}\left(\xi_{i}+\xi_{i}^{*}\right)\text{.} \end{array}$$

In formula (2), *C* is the penalty parameter and (*C* > 0) is the sample penalty beyond *ε*. The constraint condition of formula (2) is as follows:

(3) $$\begin{array}{c} \left\{\begin{array}{c} y_{i}-w∙\varphi \left(x_{i}\right)-b\leq \xi_{i}+\varepsilon \text{,}\\ w.\varphi \left(x_{i}\right)+b-y_{i}\leq \varepsilon +\xi_{i}^{*}\text{,}\\ \xi_{i}\geq 0,\xi_{i}^{*}\geq 0\text{.} \end{array}\right. \end{array}$$

Aiming at the convex quadratic optimization problem, the Lagrange function is introduced for partial differentiation so as to obtain the dual form of the optimization target and the maximization function.

(4) $$\begin{array}{c} W\left(\alpha ,\alpha^{*}\right)=-\frac{1}{2}\overset{n}{\underset{i,j=1}{\sum }}\left(\alpha_{i}-\alpha_{i}^{*}\right)\left(\alpha_{j}-\alpha_{j}^{*}\right)\left(x_{i}∙x_{j}\right)+\overset{n}{\underset{i=1}{\sum }}\left(\alpha_{i}-\alpha_{i}^{*}\right)y_{i}-\overset{n}{\underset{i=1}{\sum }}\left(\alpha_{i}-\alpha_{i}^{*}\right)\varepsilon \text{.} \end{array}$$

The constraint condition is as follows:

(5) $$\begin{array}{c} \left\{\begin{array}{c} \overset{n}{\underset{i=1}{\sum }}\left(\alpha_{i}+\alpha_{i}^{*}\right)=0\text{,}\\ 0\leq \alpha_{i},\alpha_{i}^{*}\leq C\text{.} \end{array}\right. \end{array}$$

**Figure 1:**
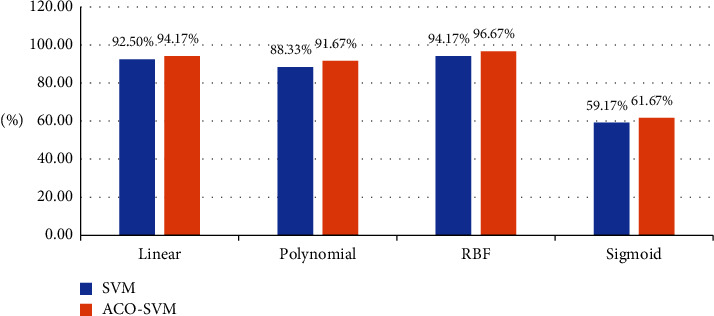
Comparison of model selection accuracy with four different kernel functions.

In formula (5), *α* and *α*^*∗*^ are Lagrange multipliers. In the dual problem, the optimization of the objective function only involves the inner product operation between training samples (*x*_*i*,_*y*_*j*_), so the inner product operation is actually carried out in higher dimensions. This operation can be realized by using the functions in the original space. According to functional theory, as long as a kernel function *K*(*x*_*i*,_*y*_*j*_) satisfies the Mercer condition, it will correspond to the inner product of the spatial transformation. SVM constructed by different kernel functions is different [18].The key point of SVM classification lies in the construction and selection of kernel functions. Appropriate kernel functions can effectively solve the problem of dimension disasters in a high-dimensional space and reduce the complexity of calculation in a high-dimensional space. There are four kernel functions commonly used in SVM: linear kernel function, polynomial kernel function, RBF kernel function, and sigmoid kernel function. When the ACO-SVM model is used, the accuracy of parameter selection and the classification time are taken as the effective indexes to evaluate the parameter model. In order to make the results more close to its real performance, a grid optimization method and cross validation of ten-fold are adopted. It can be found from Figure 1 that the ACO-SVM model has the highest efficiency when an RBF kernel function is adopted, and no matter which kernel function is adopted, its accuracy is improved compared with SVM classification directly. Specifically, when linear, polynomial, RBF, and sigmoid kernel functions are adopted, accuracy increases by 1.6%, 3.34%, 2.5%, and 2.5%, respectively. As can be seen from Table 1, in terms of selection efficiency, no matter which kernel function is used, the classification efficiency of the ACO-SVM model is significantly improved compared with that of direct selection, indicating that the ACO-SVM selection model is very effective for slope displacement prediction. Its expression is as follows:

(6) $$\begin{array}{c} K\left(x_{i,}y_{j}\right)=\exp\left(-\gamma {\left\|x_{i}-y_{j}\right\|}^{2}\right)\text{.} \end{array}$$

Then, formula (4) can be converted to the following formula, which is expressed as follows:

(7) $$\begin{array}{c} W\left(\alpha ,\alpha^{*}\right)=-\frac{1}{2}\overset{n}{\underset{i,j=1}{\sum }}\left(\alpha_{i}-\alpha_{i}^{*}\right)\left(\alpha_{j}-\alpha_{j}^{*}\right)K\left(x_{i}∙x_{j}\right)+\overset{n}{\underset{i=1}{\sum }}\left(\alpha_{i}-\alpha_{i}^{*}\right)y_{i}-\overset{n}{\underset{i=1}{\sum }}\left(\alpha_{i}-\alpha_{i}^{*}\right)\varepsilon \text{,} \end{array}$$

*w*=∑_*i*=1_^*n*^(*α*_*i*_ − *α*_*i*_^*∗*^)*φ*(*x*_*i*_), and solving the convex quadratic programming problem to nonlinear mapping is represented by the following formula:

(8) $$\begin{array}{c} y\left(x\right)=\overset{n}{\underset{i=1}{\sum }}\left(\alpha_{i}-\alpha_{i}^{*}\right)K\left(x_{i}∙x_{j}\right)+b\text{.} \end{array}$$

From (5) and (6), it can be seen that the parameters in penalty functions *C* and *K*(*x*_*i*_∙*x*_*j*_) kernel functions in support vector machines have great influence on the generalization ability and calculation efficiency of the algorithm. Generally, it is blind and inefficient to determine two parameters manually. Based on the situation, the paper uses the ant colony algorithm (ACO) to search for parameters to find the optimal support vector machine parameters [14].

### 2.3. Continuous Domain's Ant Colony Algorithm

The ant colony algorithm (ACO) is a new intelligent optimization algorithm proposed by Italian scholar Dorigo in the early 1990s [19]. The algorithm was first used to solve the discreteness optimization problem. The optimization of support vector machine parameters is to solve the problem of continuous domains. In this paper, the ant colony algorithm is improved by optimizing parameters of continuous domain model, and the model parameters of the continuous domain are optimized. The key factors of the ant colony algorithm are the movement rule and pheromone update. The ant colony searches for positive feedback of volatile accumulation of pheromone and selects the optimal path [20].

Suppose the objective function of the continuous field is as follows:

(9) $$\begin{array}{c} \left\{\begin{array}{c} \min\;Q=f\left(x\right),x=\left(x_{1},x_{2},\cdots ,x_{D}\right)\text{,}\\ x_{b}^{1}\leq x_{b}\leq x_{b}^{u};b=1,2,\cdots ,D\text{.} \end{array}\right. \end{array}$$

In the formula, *xu*/*b* and *x*1/*b* are the upper and lower limits of the independent variable *x*_*b*_ and *D* is the number of independent variables. The search optimization steps for formula (9) by using the ant colony algorithm are as follows:

(1) Ant colony initialization. The ant colony size is set as *N*, the number of cycles is *K*, and the ant colony is randomly distributed in the optimization space [21]. As the starting point for each ant to search, the continuous domain is discretized into a number of intervals, and the length matrix of each variable quantum interval is expressed as formula:
  (10) $$\begin{array}{c} Len\left(b\right)=\frac{x_{b}^{1}-x_{b}^{u}}{N} \end{array}$$
According to the current location situation of ants and different types of optimization goals, we make sure the initial pheromone concentration vector Τ(*i*) of the ant *i* is as follows:
  (11) $$\begin{array}{c} Τ\left(i\right)=\exp\left(-f\left(x_{i}\right)\right)\text{.} \end{array}$$
In formula (11), *x*_*i*_=*xi*/1, *xi*/2, ⋯, *xi*/*j*, ⋯, *xi*/*D*, *i*=1,2, ⋯, *N*, *N* is the initial position of the ant *i*. From formula (10), it can be known that the target function value *f*(*x*_*i*_) is smaller, and more pheromones are left by the position *x*_*i*_ of the ant *i*.
(2) Movement rules of ant colony: When all ants complete searching, they will start the next searching according to the corresponding movement rules. In the paper, the dynamic global selection factor and the dynamic volatile factor are introduced to improve the global searching ability [22].
The basic rules of searching: After the ant colony completes a cycle, one ant will find the optimal solution of the cycle, namely, the head ant, and its position is *x* leader. The other ants in the next cycle will use the head ant position as the target for transfer searching, which is called global search. The leader with the optimal solution is randomly searched in the neighbourhood to obtain a better solution, which is called local searching [23]. The transfer probability of the ant *i* at the position *x*_*i*_(*i* = 1, 2,…, *N*, *i* ≠ leader) to the position *x*_leader_ of the head ant is *P*(*i*):
  (12) $$\begin{array}{c} P\left(i\right)=\frac{\left.\exp\;\left(T\left(leader\right)\right.-T\left(i\right.\right)}{\exp\;\left(T\left(leader\right)\right)}\text{.} \end{array}$$
In formula (12), *τ*(leader) is a *t* pheromone concentration of the first ant's location and *τ*(*i*) is a pheromone concentration of the location of the ant *i*.
In the global searching, the selection factor of the dynamic global *P*_0_ is introduced into the step size transferred from the ant *i* to the optimal solution position *x*_leader_. The specific expression is
  (13) $$\begin{array}{c} x_{i}=\left\{\begin{array}{c} \left(1-\lambda \right)x_{i}+\lambda x_{leader}P\left(i\right)<P_{0}\text{,}\\ x_{i}+ran\;d\left(-1,1\right)\times LenP\left(i\right)\geq P_{0}\text{.} \end{array}\right. \end{array}$$
The local searching is a random searching in the *x*_leader_ neighborhood of the ant leader. Let the new search location be *x*_temp_. If *x*_temp_ is better than *x*_leader_, we replace *x*_leader_ with *x*_temp_. Otherwise, we keep the original location. In order to obtain the optimal solution of later fine searching, the step size is introduced to update a parameter *w*, so the searching step size decreases with the increase of iteration times.
Specifically, it is expressed as follows:
  (14) $$\begin{array}{c} x_{leader}=\left\{\begin{array}{c} x_{temp\;},T\left(leader\right)<T\left(temp\right)\text{,}\\ x_{leader},else\text{.} \end{array}\right. \end{array}$$
In formula (14), *τ*(temp) is a pheromone concentration of the ant location *x*_temp_.
  (15) $$\begin{array}{c} x_{temp}=\left\{\begin{array}{c} x_{leader}+w\times step,ran\;d\left(1\right)<0.5,\\ x_{leader\;}-w\times step,else, \end{array}\right. \end{array}$$
In formula (15), step = 0.1 × rand(*D*, *N*, *K*) is the local search step size, and the *w* step size updating rule is as follows:
  (16) $$\begin{array}{c} w=w_{\max}-\left(w_{\max}-w_{\min}\right)\frac{k}{K}\text{.} \end{array}$$
In formula (16), *w*_max_ an d *w*_min_ are the initial setting values, generally *w*_min_∈(0.2, 0.8) and *w*_max_∈(1.2, 1.4); *k* is an iteration number of the current ant colony; *K* is the maximum iteration number of the ant colony.
(3) Pheromone updating rules. In the completion of global searching and local searching, ants will update the pheromone *τ*(*i*) of the location of the ant *i*. The pheromone updating rules can be expressed as follows:
  (17) $$\begin{array}{c} T\left(i\right)=\left(1-\rho \right)T\left(i\right)+\Delta T\left(i\right)\text{.} \end{array}$$
In formula (17), Δ*T*(*i*) is a pheromone increment and Δ*T*(*i*)=exp (−*f*(*x*_*i*_)). *ρ* is a pheromone volatilization factor (*ρ*∈(0,1)) and, and the dynamic change is first small and then large with the number of iterations. The reason is to expand the global searching capability in the early stage and accelerate the convergence rate in the later stage.

### 2.4. Slope Displacement Prediction

The comprehensive prediction model of the slope displacement needs to consider emphatically:(1) how to choose the influencing factors of the slope displacement and the corresponding relationship between them and the slope displacement; (2) how to realize the time series decomposition of the slope displacement and whether the decomposition quantity has physical significance; (3) construction of an efficient and reliable slope displacement prediction model [24]. The combination of three methods can effectively improve the accuracy of slope displacement prediction. In order to obtain the random term of the slope displacement, the commonly used methods include empirical mode decomposition (EMD), ensemble empirical mode decomposition (EEMD), and wavelet analysis. However, their decomposition components are usually more than five, and the physical meaning represented by each component is difficult to understand [25]. To solve the above problems, SVM is used to extract the displacement component of slope random terms. SVM can achieve adaptive decomposition according to the data scale and obtain the displacement of the trend term, periodic term, and random term by setting the modal component, which has the advantages of high decomposition accuracy, strong robustness, and clear physical meaning. Therefore, SVM can be combined with time sequence analysis to achieve effective extraction of a random term displacement. In this process, the optimal decomposition parameters can be further determined by introducing an ACO algorithm. The double exponential smoothing (DES) method was used to predict the trend term displacement [26]. As a special weighted moving average method, DES is more suitable for the prediction of time series with a certain trend. Its characteristic is that the weight of the latest data is higher than that of early data, and the factor of this weight decreases exponentially with aging of data, taking into account the timeliness of landslide displacement data.

The abovementioned ant colony algorithm is used to optimize the parameter searching in the kernel functions of penalty functions *C* and *K*(*x*_*i*_, *x*_*j*_) in the support vector machine [27]. First, the objective function is determined as follows:

(18) $$\begin{array}{c} \left\{\begin{array}{c} \min\;f\left(C,\gamma \right)=\sqrt{\frac{1}{n}\overset{n}{\underset{i=1}{\sum }}\left(z_{i}-{\hat{z}}_{i}\right),}\\ C\in \left[C_{\min},C_{\max}\right],\gamma =\left[\gamma_{\min},\gamma_{\max}\right]\text{.} \end{array}\right. \end{array}$$

In formula (18), *z*_*i*_ is the measured displacement of its sample and ${\hat{z}}_{i}$ is a predictive value for samples. In formula (8), *n* is the total number of training samples. Due to the monitoring displacement, {*x*_1_, *x*_2_,…, *x*_n_} is a time series, and its phase space is reconstructed to find the *i* + *p* time displacement sequence value and the sequence value (*x*_i_, *x*_i+1_ ...*x*_i+p−1_) at the previous *p* months, namely, *x*_*i*+*p*_ = *g*(*x*_i_, *x*_i+1_,*x*_i+p−1_). *p* is the number of historical steps, and *g* is the nonlinear mapping function. According to SVM theory, the nonlinear relation of displacement time series can be described as follows:

(19) $$\begin{array}{c} \hat{z}\left(x_{m+n}\right)=\overset{n-p}{\underset{i=1}{\sum }}\left(\alpha_{i}-\alpha_{i}^{*}\right)-K\left(x_{m+n},x_{i+p}\right)+b\text{.} \end{array}$$

In formula (19), $\hat{z}\left(x_{m+n}\right)$ represents the predicted displacement at time *m* + *n*; *x*_*m*+*n*_ represents the measured displacement time series value *x* at *p* moments before time *m* + *n x*_*m*+*n*_ = (*x*_m+*n*−*p*_, *x*_*m*+*n*−*p*+1_, *…*, *x*_*m+n*−1_); *x*_*i*+*p*_ represents the measured displacement time series value *x*_*i*+*p*_ = (*x*_i_, *x*_*i*+1_,…, *x*_*i*+*p*−1_) at *p* moments before the (*i* + *p*) moment. It should be noted that the predicted value is used as the training and learning sample of the next prediction. After all, the predicted value is not measured and has a certain range of application. In the actual engineering process, the prediction model can be updated in real time with the acquisition of the monitoring value [16]. In the algorithm model, ACO optimizes the parameters in SVM. The main idea is to search for the smallest set of parameters by defining a set of parameters (*C*, *γ*) in the domain as the position vector of ants so that the predicted value can be closer to the monitored value. The specific steps are as follows:

An ACO-SVM prediction model was built based on the deep learning toolbox framework of MATLAB2020a software and completed on the computer with Intel Core I5–9400F CPU and 16G RAM.

1. According to the monitored displacement data, the historical step size *p* and the predicted step size *m* are normalized, learning samples and test samples are established, and the dataset is normalized by min-max.
2. We set the initialization of the system, including ant colony size *N*, cycle iteration times *K*, the value range of the parameter *C* and *γ* to be optimized, the number of steps, and the location of the ant. Each position value corresponds to a set of parameters (*C*, *γ*).
3. The optimization support vector machine (SVM) learning prediction model is established based on the theory in Section 2 of this paper, and the corresponding objective function of each ant individual is calculated, as shown in formula (18). Global searching and local searching are conducted, and pheromones are updated to determine the optimal solution.
4. First, we determine the appropriate number of iterations through multiple iterations and then judge whether the value of the objective function meets the conditions. If the conditions are met, the optimization is finished, and the optimal parameters *C* and *γ* are output. Otherwise, we return to step (3).
5. The optimized parameters *C* and *γ* are used to establish a support vector machine (SVM) prediction model to carry out rolling prediction of displacement time series and fulfil engineering prediction requirements.

**Figure 2:**
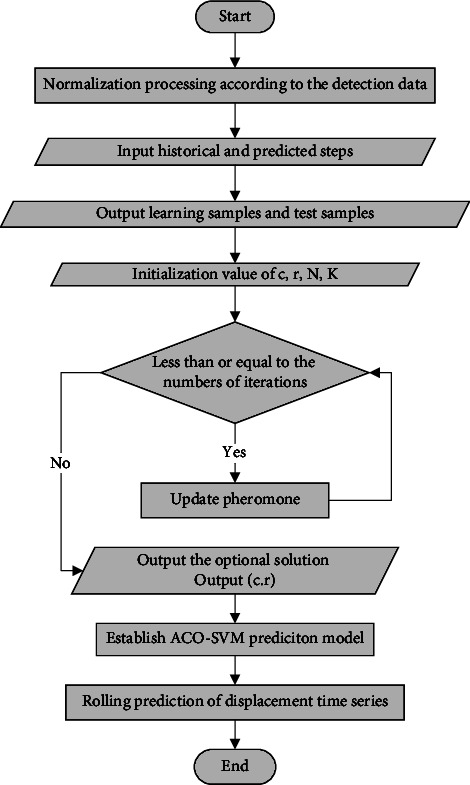
Flowchart of ACO-SVM optimization for prediction of the slope displacement.

The process of optimization ACO-SVM for prediction of the slope displacement is shown in Figure 2.

### 2.5. Model Evaluation Index

The coefficient of determination (*R*^2^) and the root mean square error (RMSE) are often used to evaluate the performance of slope displacement prediction models. Therefore, the performance of the prediction model is analysed based on these indicators, which are defined as follows:

(20) $$\begin{array}{c} R^{2}=1-\frac{\sum_{t=1}^{N}{\left(y_{t}-\hat{y_{t}}\right)}^{2}}{\sum_{t=1}^{N}{\left(y_{t}-\bar{y_{t}}\right)}^{2}}\text{,} \end{array}$$

(21) $$\begin{array}{c} RMSE=\sqrt{\frac{1}{N}\overset{N}{\underset{t=1}{\sum }}{\left(y_{t}-\hat{y_{t}}\right)}^{2}.} \end{array}$$

In formula (20) and (21), *t* denotes the time, N denotes the predicted time, *y*_*t*_denotes the actual observed value of the landslide displacement, $\hat{y_{t}}$ denotes the predicted value of the landslide displacement in our model, and $\bar{y_{t}}$ denotes the average of the observed values of the displacement in the predicted time. The value range of RMSE is [0, *∞*], and the smaller the value , the stronger the model fitting ability. The value of *R*^2^ is usually [0, 1], and the larger the value, the better the surface model fitting degree. When the value of RMSE is smaller and the value of *R*^2^ is larger, the prediction effect of the model is better.

## 3. Algorithm Application

### 3.1. Shallow Deformation Prediction of the Left Bank Slope of the Hydropower Station

In the paper, the improved ant colony algorithm is used to optimize the support vector machine (ACO-SVM) to predict the displacement of the monitoring displacement sequence of the slope in reference 10. Monitoring data are the orifice displacement of the M4-7 multipoint displacement meter (elevation 1886 m) of the left bank cable crane platform slope at the exposed part of f42-9 fault[10].

The learning samples are shown in Table 2 to verify the accuracy of the ACO-SVM prediction model.

**Figure 3:**
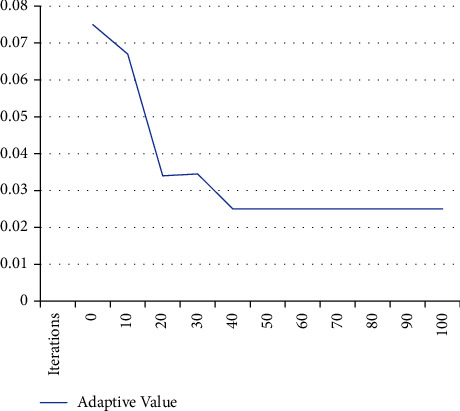
Performance evaluation of the ACO feature selection algorithm.

In this paper, the improved ACO algorithm is used for feature selection and combined with the SVM data model, and comparative simulation experiments are carried out. In the experimental steps, the history of the selected feature set is set to 10, the prediction step is set to 1, the maximum number of iterations is set to 100, and the classification error rate is taken as the adaptive value of the fitness function. The performance of the feature selection algorithm is evaluated by the support vector machine (SVM) classifier (mesh optimization method is used to calculate the optimal parameter values) and the adaptive values obtained by cross-validation. The lower the adaptive value, the better the performance of the feature selection algorithm. The relation between the ACO iteration number and adaptive value is shown in Figure 3. The optimal result is 0.025, and the earliest algebra value of the optimal result is 39. In order to reduce the computational overhead without significantly reducing the optimization effect, it is appropriate to set the maximum iteration number to 50. By calculating the evaluation indexes *R*^2^ and RMSE of the SVM data model, the prediction result of the periodic displacement can be obtained. With the increase of the training set data, the prediction accuracy of the model is also improved. For periodic datasets, ACO-SVM can achieve the best effect for random datasets. The model parameters (*C*, *γ*) obtained by the ACO optimization method proposed in this paper are 20.305 and 2.514, respectively. The prediction results of the ACO-SVM model are shown in Table 3. Compared with the prediction results of the improved SVM (hybrid kernel least-squares support vector machine) in literature [14], the accuracy of the ACO-SVM prediction is better than that of the improved SVM except for a few points. The maximum relative error of the ACO-SVM prediction is 2.23%, less than 3.19% of the improved SVM, which proves the correctness of the ACO-SVM prediction model proposed in the paper.

### 3.2. Prediction Grey Model for the Slope Displacement in the Hydropower Station

The first-level hydropower project of HuaGuangTan is located in the middle and lower reaches of the Juxi River, Lin'an city, Zhejiang province. The first-level plant is located on the right bank of Juxi, and the overall flow direction of the nearby rivers is 120°∼130°. The slope behind the plant was fully developed, and the gully direction is 40°∼50°, which is nearly orthogonal to the Juxi River. The upper and lower reaches of the workshop have a cutting depth of 50∼70 m, forming a long and narrow ridge facing the air and spreading in the northeast direction. The surface of the slope body is quaternary colluvial deposit, the middle part is the broken rock body with strong unloading action, and the lower part is fresh Jurassic fused tuff. During the heavy rainfall from May to June 2018, subsidence and external bulge occurred in 250 raceways of the plant area, and multiple tensile cracks were found in the middle and upper part of the slope body during a subsequent supplementary survey. In order to grasp the law and development trend of slope deformation, a safety monitoring system for slope deformation was established. In the established slope surface observation system, a total of 14 surface displacement observation points are arranged, and the number of displacement observation points is 1# to 14# according to elevation.

In the paper, the horizontal displacement data of measuring point 7# on the direction of the main slide are selected to predict and analyse the slope deformation. Through trial calculation, the optimal historical step *p* is 4, the predicted step *m* is 1, the ant colony size is 200, the total number of iterations is 50, and the optimal objective function value is *f* < 1 × 10^−6^. 30 displacement timing series monitored from the measuring point 7#, 2019-01-20 to 2019-08-11 (as shown in Table 4) were constructed into 26 training samples, and the next 10 displacement timing series were predicted with a time interval. The new prediction value was added to the training sample, the first training sample was deleted and the number of samples was kept unchanged, then the next prediction was performed, and so on.

Using ACO-SVM model research on the monitoring data in Table 3 for training and prediction analysis, at the same time in order to compare the accuracy of the models respectively, the traditional SVM, GA to optimize SVM and PSO to optimize SVM to forecast the monitoring data analysis and comparison of model parameter optimization (*C*, *γ*) is shown in Table 5, and the corresponding prediction results are shown in Table 6.

**Figure 4:**
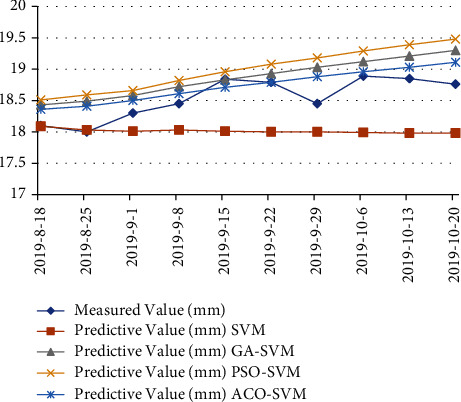
Comparison diagram between the measured value and predicted value (four algorithms).

**Figure 5:**
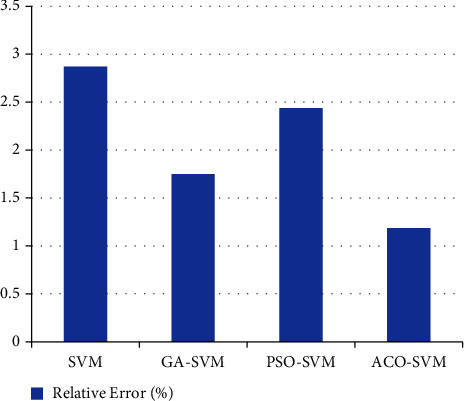
Comparison diagram of the relative error between ACO-SVM and three algorithms.

According to Table 5, it can be seen that most of the prediction results of the ACO-SVM model are better than those of the other three prediction models in Figure 4, with higher prediction accuracy, the maximum relative error is 2.31%, which is smaller than the maximum relative error of the other three models. The average relative error rate of the ACO-SVM algorithm is 1.19%, which is obviously lower than that of the other three algorithms in Figure 5. The traditional SVM model has high short-term prediction accuracy but weak generalization ability and large prediction error. The improved ACO-SVM model overcomes the problems of generalization ability and prediction error. The generalization ability of the three SVM models using the optimization algorithm is better than that of the traditional SVM model.

**Figure 6:**
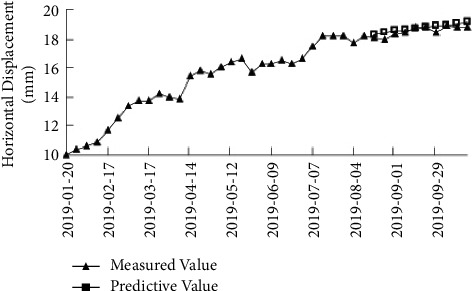
Comparison between the measured value and the predicted value for monitoring point 7#.

By comparing the predicted value of the ACO-SVM model with the measured value in Figure 6, it can be seen from the change law that the slope displacement changes little at the later stage, the deformation trend is gentle, and the displacement tends to converge, which proves that the slope is relatively stable. The measured value of the slope displacement at the later stage also verifies that the displacement tends to converge, so the predicted result meets the actual demand of the project.

## 4. Conclusions

It is of great significance for engineering construction and safe operation to predict the future deformation trend of the slope based on displacement monitoring data. In view of the influence of model parameters (*C*, *γ*) of the support vector machine (SVM) on prediction accuracy and generalization ability, in order to avoid the blindness of artificial parameter selection, the paper proposed the improved ant colony algorithm to optimize SVM, search for optimal parameters, and apply the model to deformation prediction of slope engineering examples. It is found that the ACO-SVM model is able to predict the slope deformation and meet the demand of engineering prediction.

In the prediction of shallow deformation of the left bank slope of a first-stage hydropower station, the accuracy of the ACO-SVM model and the hybrid kernel least-squares SVM model is similar, which is more accurate than the traditional SVM model, indicating the accuracy of the ACO-SVM model.

The ACO-SVM model has higher prediction accuracy and stronger generalization ability than the genetic algorithm and particle swarm optimization to optimize SVM prediction results.

The comparison between the predicted value of the improved ACO-SVM model and the measured value shows that according to its variation law, the slope displacement changes little in the late stage, the deformation trend is gentle, and the displacement tends to converge, which proves that the slope is relatively stable. The measured value of the slope displacement in the later period also verifies that the displacement tends to converge, so the predicted results meet the actual engineering needs.

The ACO-SVM also has some shortcomings. For example, the searching time of this algorithm is longer than that of other optimization algorithms, which is a process of balancing accuracy and time consumption in optimization problems, which needs further research.

## Figures and Tables

**Table 1:** Comparison of model classification efficiency using four different kernel functions.

Classification model	Time (s)			
	Linear	Polynomial	RBF	Sigmoid
SVM	183.39	148.22	170.48	159.21
ACO-SVM	7.37	7.6	7.17	7.01

**Table 2:** Training data of multipoint extensometer M4-7.

ID	Measured value (mm)	ID	Measured value (mm)	ID	Measured value (mm)
1	7.65	11	10.55	21	12.21
2	8.04	12	10.70	22	12.26
3	8.28	13	10.83	23	12.18
4	8.66	14	10.83	24	12.53
5	9.04	15	11.34	25	12.56
6	9.38	16	11.20	26	12.70
7	9.93	17	11.44	27	12.72
8	10.04	18	11.35	28	12.85
9	10.15	19	11.59	29	12.88
10	10.34	20	11.71	30	13.11

**Table 3:** Comparison of prediction results among different models for extensometer M4-7.

ID	Measured value/mm	Predicted value (mm)			Relative error (%)		
		SVM	Improved SVM	ACO-SVM	SVM	Improved SVM	ACO-SVM
31	12.83	13.27	13.02	13.01	3.40	1.45	1.41
32	13.01	13.30	13.05	13.00	2.20	0.55	0.57
33	12.76	13.27	12.99	12.91	4.03	1.79	1.17
34	12.86	13.30	13.06	12.99	3.42	1.56	1.02
35	13.14	12.50	12.72	12.74	4.91	3.19	2.23
36	13.13	12.66	12.96	12.94	3.62	1.40	1.47
37	13.01	13.28	13.01	12.92	2.09	0.73	0.72

**Table 4:** Horizontal resultant displacement of monitoring point 7#.

Date	Measured values (mm)	Date	Measured values (mm)	Date	Measured values (mm)
2019-01-20	10.06	2019-03-31	14.01	2019-06-09	16.27
2019-01-27	10.34	2019-04-07	13.83	2019-06-16	16.52
2019-02-03	10.62	2019-04-14	15.42	2019-06-23	16.28
2019-02-10	10.90	2019-04-21	15.75	2019-06-30	16.63
2019-02-17	11.73	2019-04-28	15.53	2019-07-07	17.50
2019-02-24	12.55	2019-05-05	16.01	2019-07-14	18.18
2019-03-03	13.38	2019-05-12	16.33	2019-07-21	18.24
2019-03-10	13.74	2019-05-19	16.65	2019-07-28	18.21
2019-03-17	13.78	2019-05-26	15.70	2019-08-04	17.68
2019-03-24	14.20	2019-06-02	16.27	2019-08-11	18.22

**Table 5:** Optimal parameters of different prediction models.

Model name	*C*	*γ*
SVM	128.00	0.001
GA-SVM	99.30	0.005
PSO-SVM	100.00	0.010
ACO-SVM	44.68	2.990

**Table 6:** Comparison of prediction results among different models for monitoring point 7#.

Date	Measured value (mm)	Predictive value (mm)				Relative error (%)%			
		SVM	GA-SVM	PSO-SVM	ACO-SVM	SVM	GA-SVM	PSO-SVM	ACO-SVM
2019-08-18	18.10	18.09	18.43	18.51	18.36	0.06	1.86	2.26	1.46
2019-08-25	18.00	18.03	18.49	18.59	18.41	0.17	2.76	3.30	2.29
2019-09-01	18.30	18.01	18.58	18.66	18.50	1.56	1.53	2.00	1.08
2019-09-08	18.45	18.03	18.72	18.82	18.61	2.30	1.42	1.96	0.85
2019-09-15	18.84	18.01	18.83	18.96	18.71	4.39	0.04	0.64	0.71
2019-09-22	18.79	18.00	18.93	19.08	18.79	4.19	0.76	1.53	0.01
2019-09-29	18.45	18.00	19.03	19.18	18.88	2.46	3.14	3.96	2.31
2019-10-06	18.89	17.99	19.12	19.29	18.96	4.76	1.24	2.09	0.36
2019-10-13	18.85	17.98	19.21	19.39	19.03	4.61	1.91	2.82	0.96
2019-10-20	18.76	17.98	19.3	19.48	19.11	4.19	2.84	3.8	1.82

## Data Availability

The data supporting the findings of this study are available within the article.
